# A Thermodynamically Consistent Approach to Molecular Simulations of Adsorption-Induced Deformation and Structural Transitions in MOFs

**DOI:** 10.1021/acs.jctc.6c01243

**Published:** 2026-10-07

**Authors:** Nicholas J. Corrente, Kaelyn Chang, Muhtasim Noor, Alexander V. Neimark

**Affiliations:** Department of Chemical and Biochemical Engineering, Rutgers University, Piscataway, New Jersey 08854, United States

## Abstract

Flexible metal–organic frameworks (MOFs) exhibit coupled adsorption-deformation behavior that cannot be captured in molecular detail by conventional rigid-framework simulations. We present an iterative hybrid GCMC/MD methodology that explicitly couples grand canonical Monte Carlo sampling of adsorbate configurations with isothermal–isobaric molecular dynamics relaxation of the framework structure, using a Metropolis acceptance criterion in the osmotic ensemble to ensure thermodynamic consistency. Applied to argon adsorption on ZIF-8 at 87.3 K, the method quantitatively reproduces the experimental stepped isotherm characteristic of the gate-opening transition between low pressure (LP) and high pressure (HP) conformations and predicts nonmonotonic strain isotherm with initial contraction (∼0.5%) at low loadings followed by expansion (∼0.7%) during gate-opening. In addition, the elastic modulus variation upon loading is calculated from the volume fluctuations. The simulations reveal and quantify the molecular mechanism of gate-opening through cooperative linker reorientation from a unimodal swing angle distribution centered at 0° to a bimodal distribution peaked at ± 23°. The proposed approach is computationally efficient, yielding converged strain and compressibility isotherms within an accessible number of iterations, and provides a general framework for predicting adsorption-induced structural transitions in flexible porous materials without *a priori* knowledge of end point structures.

## Graphical abstract

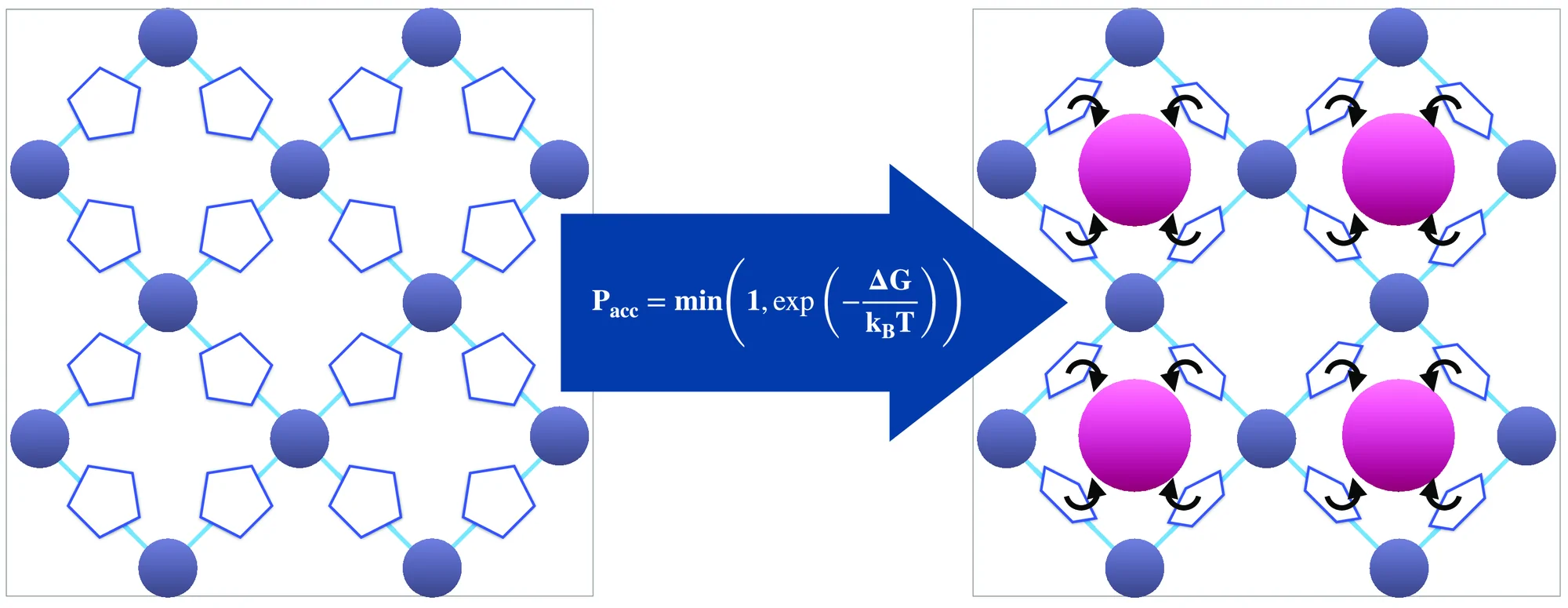

## Introduction

Metal–organic frameworks (MOFs) have emerged as a versatile class of porous materials with applications spanning gas storage, separation, and catalysis.^1–7^ MOFs exhibit structural flexibility, wherein framework deformation occurs in response to external stimuli such as guest adsorption, temperature, or pressure.^5,8–13^ Framework flexibility can manifest as elastic expansion and contraction, as well as structural transformations like breathing transitions between distinct framework conformations and gate-opening transitions with abrupt pore aperture enlargement due to linker rotation or folding. It is well documented that structural flexibility significantly affects MOF adsorption properties and enables the design of compliant molecular sieves, membranes, sensors, and actuators^14–17^ often producing characteristic stepped adsorption isotherms.^18,19^ Rational design of flexible MOF structures for specific applications requires synergistic coupling of their adsorption and mechanical properties. In this paper, we propose an advanced molecular simulation method for quantitatively predicting adsorption and strain isotherms, as well as framework compressibility. The method is illustrated drawing on an example of the ZIF-8 MOF that exhibits prominent framework flexibility with the gate opening transition.

Zeolitic imidazolate frameworks (ZIFs) represent a distinctive subclass of MOFs, in which the metal-imidazolate-metal bridging angle closely mimics the Si–O–Si angle in aluminosilicate zeolites, enabling ZIFs to adopt topologies analogous to classical zeolite skeletons.^20^ Among the numerous ZIF structures reported, ZIF-8 has emerged as a prototypical system owing to its exceptional thermal stability, chemical resistance, and porosity with the Brunauer–Emmett–Teller (BET) surface area >1800 m^2^/g.^20^

ZIF-8 crystallizes in the cubic sodalite topology, featuring large cavities with diameters of ∼1.16 nm interconnected through six-membered ring windows with crystallographic apertures of only 0.34 nm, see Figure 1. This narrow window size is significant because it lies below the kinetic diameters of many industrially relevant molecules, yet experimental observations demonstrate that molecules too large to pass through these apertures readily adsorb and diffuse within the framework. Fairen-Jimenez et al.^21^ and Ania et al.^22^ demonstrated unexpected adsorption of nitrogen and argon despite their kinetic diameters exceeding the crystallographic pore aperture, while Kolokolov et al.^23^ studied benzene adsorption where the size mismatch was even more dramatic. This apparent contradiction points to the importance of framework flexibility, specifically the ability of the 2-methylimidazolate (MeIM) linkers to rotate due to thermal fluctuations and undergo dynamic reorientations that enlarge the window apertures. Moreover, high-pressure crystallographic studies by Moggach et al.^24^ and in situ diffraction measurements by Fairen-Jimenez et al.^21^ established that mechanical compression or gas adsorption induces the transition with linker reorientation of approximately 20–25° from the low pressure (LP) to high pressure (HP) framework configuration with increased pore volume. Subsequently, Hobday et al.^25^ combined high-pressure single-crystal diffraction in a diamond anvil cell with GCMC and periodic DFT calculations, using liquified Ar, CH_4_, N_2_, and O_2_ as both pressure-transmitting media and adsorbates, and resolved six symmetry-independent adsorption sites in the HP phase together with linker rotation accompanying the transition. Their calculations quantified the energetic balance underlying gate-opening: the DFT energy penalty for linker rotation is offset by the gain in host–guest interaction energy in the more open HP framework. This gate opening transition gives rise to characteristic steps on adsorption isotherms of various gases.^21,22,26^ Due to the inherent flexibility of linkers, which enlarge window apertures and enable increased uptake, ZIF-8 is one of the most actively used MOFs for precise molecular sieving gas separations and storage.^27–29^

**Figure 1:**
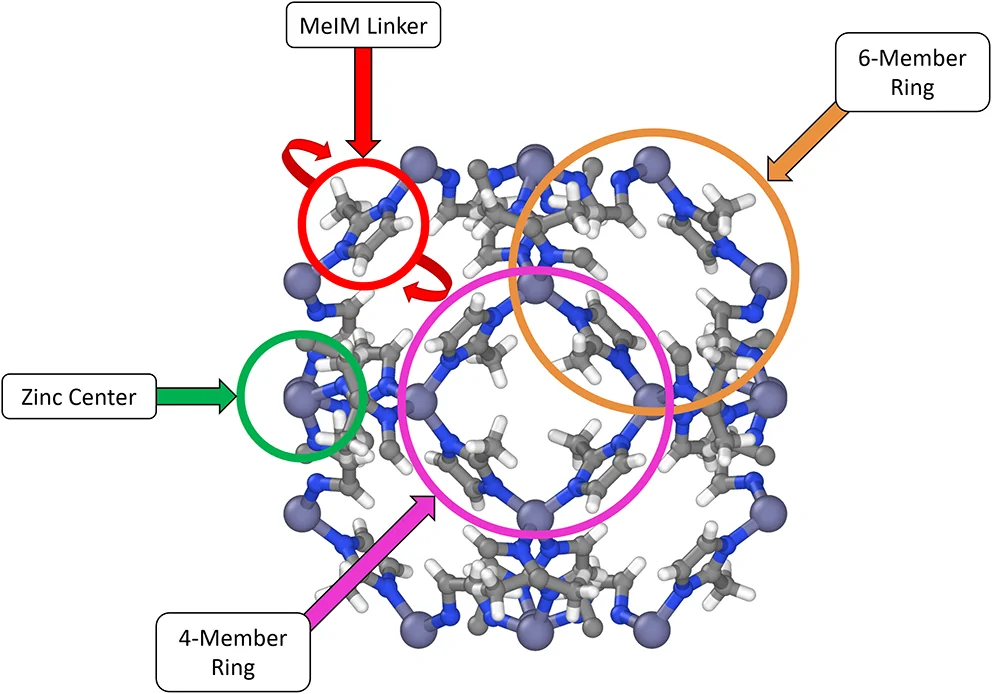
Crystallographic structure of the ZIF-8 sodalite cage in LP conformation.

Earlier computational studies of ZIF-8 employed conventional molecular simulation approaches that treat the framework as rigid, typically performing grand canonical Monte Carlo (GCMC) calculations with fixed atomic positions. Fairen-Jimenez et al.^21,30^ performed GCMC simulation of N2 adsorption on the rigid crystallographic LP and HP structures of ZIF-8 and found good agreement with the experimental data before and after the gate opening transition. Tanaka et al.^31^ elucidated the mechanics of ZIF-8 gate opening by constructing GCMC and gauge cell MC^32,33^ Ar isotherms on a set of framework structures with varying linker swing angles (θ_IM_). By comparing with the experimental data, the authors concluded that the gate opening occurs at the angle position of θ_IM_ = 25.5°.

While computationally efficient, rigid-framework MC simulations do not capture the molecular mechanisms of framework atom motion and adsorption-induced structural transitions that can be done with either *ab initio* molecular dynamics (MD),^34,35^ or classical MD with flexible force fields,.^36–38^ While these methods provide important mechanistic insights, they either require computationally expensive quantum calculations or ambiguous force fields, which are known to produce distinct results.^39^

Thermodynamic conditions of adsorption on flexible structures correspond to the osmotic ensemble^40–43^ that considers a solid–fluid system at constant gas chemical potential, external pressure, and temperature allowing for the variations of the framework volume and atoms positions. The equilibrium states correspond to the minima of the osmotic potential, Ω_os_(*N*_*f*_, μ,*P*,*T*), where the number of framework particles, *N*_*f*_, is fixed. Implementation of osmotic ensemble MC schemes with framework atom translational moves and volume change trial steps is in general impractical for complex MOF structures.^44^ Zhao et al.^45^ suggested an osmotic molecular dynamics (OMD) simulation scheme with trial insertions and deletion of guest particles. This method was applied to study breathing transitions in MIL-53 and DAT-49 MOFs and water adsorption on CAU-23 MOF.^45,46^

A practical, yet approximate approach, is to avoid the volume change trial steps and introduce an order parameter, like the framework volume and linker angle, construct a set of MD equilibrated empty frameworks of different order parameters (like volume or linker angle), simulate the MC isotherms in these frameworks, and calculate the respective osmotic potentials as functions of the order parameter at fixed pressures. Then the adsorption and strain isotherms on the flexible framework are constructed from the positions of the osmotic potential minima.^31,47^

An efficient strategy couples GCMC adsorption sampling with MD structural relaxation. Ghoufi and Maurin^43^ pioneered a hybrid osmotic Monte Carlo (HOMC) scheme, in which a short anisotropic NPT-MD segment is embedded as one of several trial moves within a single Markov chain sampling the osmotic ensemble (fixed *N*_*f*_, μ,*P*,*T*), replacing the classical volume-change move of NPT-MC. Because the MD segment competes stochastically with insertion, deletion, translation, and rotation moves in the same chain, rigorous acceptance requires explicit homogenization of the MC and MD partition functions. It is worth noting that the HOMC scheme has been implemented in RASPA software.^48–51^

Beyond HOMC, various iterative protocols^52–59^ alternate longer GCMC and MD stages sequentially, allowing mutual equilibration of adsorbate loading and framework configuration, but do so without imposing a formal acceptance criterion in the osmotic ensemble, relying instead on empirical convergence of loading and volume. A recent review by Rogge et al.^19^ surveys these hybrid MC/MD approaches for flexible MOFs, distinguishing methods that sample the full osmotic ensemble from those restricted to constant-volume subensembles, and proposed a related variant in which MD is retained as a trial move within a single Markov chain but acceptance is evaluated over a window of the MD trajectory.

Zhang et al.^52^ implemented a hybrid scheme with alternating Gibbs ensemble MC and NPT-MD steps for simulating gate opening in ZIF-8 during nitrogen adsorption. More recently, our group developed hybrid GCMC/MD protocols for modeling adsorption-induced deformation in disordered carbons^60^ and IRMOF-1 ^61^ by employing extended GCMC equilibration followed by NPT-MD relaxation. However, these iterative schemes do not explicitly enforce thermodynamic consistency through acceptance criteria in the osmotic ensemble for the transitions from the MD to MC substages during iterations.

Yu et al.^62^ performed a comprehensive assessment of an iterative hybrid GCMC/NPT-MD strategy for seven MOF structures. The authors compared the results of their flexible simulations with adsorption performed in rigid frameworks as well as results from a flexible snapshot method, in which GCMC is performed in snapshots from MD simulations of the empty frameworks in both NPT and NVT, none of which account for adsorbate-induced deformation. The authors found that the loading at 10 bar from rigid and hybrid GCMC/MD simulations differed by more than 25% in roughly half the cases. It is worth noting that their CO_2_ isotherms at 300 K on ZIF-8 (Figure 1c of ref. 62) show an insignificant difference between the flexible and rigid simulations, which is understandable since CO_2_ at these conditions does not trigger gate opening and the framework response is confined to thermal fluctuations about the LP structure.

Despite their widespread use, the fundamental validity of hybrid MC/MD schemes for flexible materials has recently been called into question. Ghoufi^63^ critically examined the ergodic properties of these methods, distinguishing between statistical ergodicity (correct sampling of ensemble distributions) and dynamical ergodicity (physical time evolution exploring all accessible phase space), noting that hybrid schemes inherently break the latter due to discontinuities introduced by insertion/deletion moves. While this distinction is important for extracting dynamical observables such as diffusion coefficients or velocity correlation functions, it does not compromise the computation of equilibrium properties, including adsorption isotherms and adsorption-induced structural transitions, provided the hybrid scheme satisfies the detailed balance in the appropriate ensemble.

Here, we present a refined hybrid iterative GCMC/NPT-MD methodology that explicitly enforces thermodynamic consistency through the Metropolis acceptance criterion in the osmotic ensemble, thereby preserving the statistical ergodicity required for reliable equilibrium sampling. We demonstrate its application with the example of argon adsorption on ZIF-8 at 87.3 K, obtaining excellent quantitative agreement with the experimental adsorption isotherm, revealing and quantifying the molecular mechanism of gate-opening through cooperative linker reorientation and predicting nonmonotonic strain isotherm and elastic modulus variation upon loading.

## Methods

The iterative simulation scheme is detailed in Figure 2. Starting from an equilibrated empty framework (50 ps NPT-MD at target temperature and pressure), each iteration proceeds through three steps: (1) GCMC simulation with rigid framework (15,000 MC moves with equal probability for insertions, deletions, and translations) to equilibrate adsorbate loading at the specified fugacity; (2) brief NVT-MD relaxation of adsorbate configuration with rigid framework (10 ps); (3) NPT-MD relaxation allowing framework flexibility (50 ps). The combination of NVT and NPT equilibrations in one substage constitutes a “volume move” in the osmotic ensemble that is subject to Metropolis acceptance

$$P_{acc}=\min\;\left(1,\exp\left(-\frac{\Delta G}{k_{B}T}\right)\right);,\Delta G=\Delta U+P\Delta V-Nk_{B}T\;\ln\;\left(\frac{V}{V_{in}}\right)$$ 1

where *P*_acc_ is the probability that the move is accepted, Δ*G* is the change in Gibbs free energy of the system, *k*_B_ is the Boltzmann constant, *T* is the set temperature, Δ*U* is the change in the potential energy, *P* is the set pressure, Δ*V* is the change in simulation cell volume, *N* is the number of molecules, *V*_in_ is the initial volume of the simulation cell, and *V* is the volume of the last frame. This acceptance criterion ensures consistency with the osmotic ensemble. Accepted configurations seed the next GCMC iteration; rejected moves revert to the last accepted structure. The cycle repeats for 1000 iterations, with volumetric and loading fluctuations monitored to assess convergence; post hoc inspection of the fluctuations confirmed convergence for all pressures studied. A representative trajectory is shown in Figure S2.

**Figure 2:**
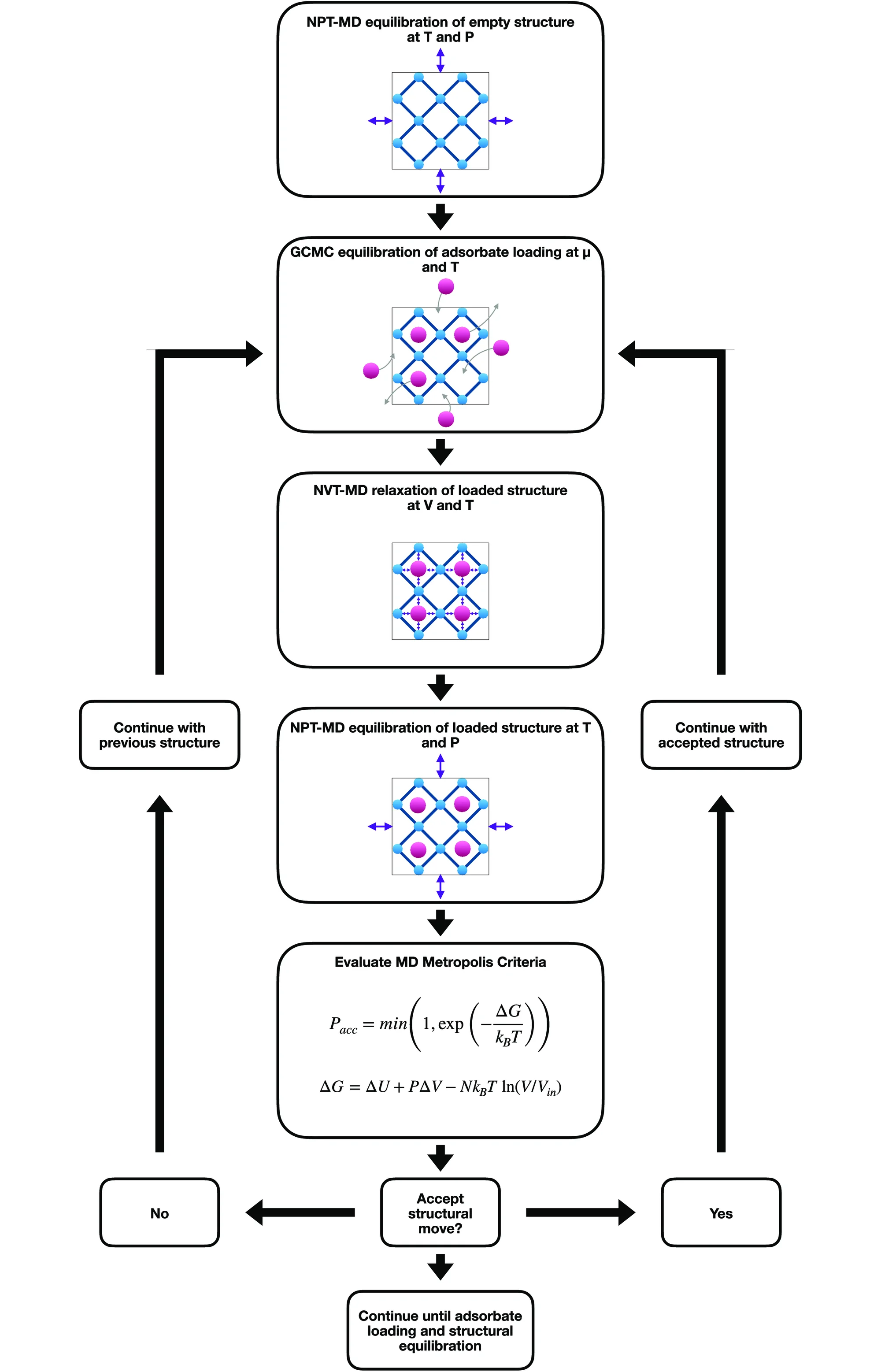
Schematic representation of the iterative GCMC/NPT-MD hybrid simulation protocol.

The form of eq 1 reflects the fact that the GCMC and MD stages are executed sequentially rather than interleaved as competing trial moves within the same Markov chain. Because adsorbate insertions and deletions are confined to the GCMC stage at fixed framework configuration and volume, and framework relaxation is confined to the MD stages at fixed adsorbate loading, the combined trialmove samples configurations are distributed according to the osmotic ensemble. The Δ*G* in eq 1 therefore contains only configurational contributions: the potential-energy change Δ*U* sampled over the NPT-MD stage, the mechanical work *P*Δ*V*, and the −*Nk*_B_ *T* ln (*V*/*V*_in_) term that accounts for the change in accessible configurational volume for the *N* adsorbate molecules upon framework deformation. Sampling within each substage is correct by construction in its respective ensemble (grand canonical at fixed *V*; isothermal–isobaric at fixed *N*), and the Metropolis criterion on the combined volume move (eq 1) ensures that the joint distribution sampled by the alternation is the osmotic ensemble. The acceptance test is evaluated on the configuration at the end of the NPT-MD segment, treating the entire segment as a single trial move; in schemes where the NPT-MD segment instead competes with insertion, deletion, and translation as a trial move within one Markov chain,^19,43^ volume is propagated by the MD barostat itself, and the acceptance criterion is formulated over the trajectory. The sequential decomposition adopted here is analogous to Gibbs sampling strategies and avoids the need to homogenize the MC and MD partition functions within a single Markov chain, which is required when MD competes with insertion/deletion as a trial move.

The efficiency of the scheme stems from the sequential alternation between GCMC (which efficiently samples adsorbate configurations) and relatively short MD trajectories (which relax framework geometry). Because the acceptance criterion is evaluated only once per GCMC/NVT/NPT cycle rather than per MD step, the cost per accepted osmotic-ensemble configuration is dominated by the MD relaxation and scales with the cost of MD segments.

Because the MD segments here constitute a single composite trial move rather than a Hamiltonian proposal in a hybrid Monte Carlo scheme, the requirements on the integrator differ from those of standard HMC. The criterion in eq 1 is configurational, and the corresponding requirement is that each MD substage preserves the invariant measure of its own ensemble: exp (−βU) at fixed *V* for the NVT segment, and exp (*βG*) for the NPT segment. The integrators used here satisfy these conditions by construction: velocity-Verlet is time-reversible, the Nosé-Hoover chain propagator of Martyna et al. (MTTK)^64^ is reversible and measure-preserving in the extended canonical phase space, and the MTTK barostat is integrated with the reversible, measure-preserving geometric integrator derived specifically for the isothermal–isobaric ensemble by Tuckerman et al.^65^

During the iterative simulations, fluctuations in the number of adsorbed particles are collected over the GCMC stages, and fluctuations in the unit cell volume are collected over the NPT-MD stages. The running averages of these fluctuations provide the adsorption *N*(*p*/*p*_0_) and strain *ε*(*p*/*p*_0_) = (⟨*V*⟩ – *V*_0_)/*V*_0_ isotherms, where *V*_0_ is the mean volume of the empty framework equilibrated at 87.3 K and *p*/*p*_0_ = 10^–8^. In addition, the isothermal compressibility of the adsorbate-loaded framework, β_*T*_, is calculated from the volume variance according to the general statistical mechanical relationship^66^

2 $$\beta_{T}=\frac{\mathrm{Var}\;\left(V\right)}{k_{B}T\;\left\langle V\right\rangle }$$

where ⟨*V*⟩ is the average volume, and *Var*(*V*) = ⟨Δ*V* ^2^⟩ is the variance in volume. Note that β_*T*_ defined in this way is the isothermal compressibility of the loaded framework at fixed (*μ, P, T*): the adsorbate number fluctuates between GCMC stages, and the volume variance in eq ([Disp-formula eq2]) is accumulated only over NPT-MD stages at the corresponding fixed loadings.

The ZIF-8 framework was modeled using a 2 × 2 × 2 supercell with force field parameters from Zhang et al.,^52^ which employ harmonic bonds and angles, CHARMM dihedrals, CVFF impropers, and Lorentz–Berthelot mixing for Lennard-Jones (LJ) interactions. Electrostatics were treated with the particle–particle-particle mesh (PPPM) with an accuracy of 10^–5^. All interactions were cut at 1.4 nm. Argon–argon interactions used standard parameters.^67^ All simulations employed LAMMPS^68^ with the Nosé-Hoover thermostat (100 fs damping) and barostat (1000 fs damping). Equations of motion were integrated with the velocity-Verlet algorithm and a time step of 1 fs; the thermostat and barostat were propagated using the time-reversible, measure-preserving factorizations of Martyna et al.^64^ and Tuckerman et al.,^65^ with the barostat in the Shinoda-Shiga-Mikami formulation^69^ and volume changes applied as an affine remapping of all atomic coordinates, with velocities redrawn at 87.3 K at the start of each NVT substage. Note that the choice of the barostat is important for proper sampling of volume fluctuations, as shown by Rogge et al.^70^ Fugacities were computed from the multiparameter equation of state^71^ via CoolProp.^72^

## Results and Discussion

The argon adsorption isotherms at 87.3 K simulated in rigid and flexible structures are presented in Figure 3 (upper panel) in comparison with the experimental isotherm^73^ from the NIST/ARPA-E database.^74^ This experimental isotherm is scaled by a multiplicative factor on the loading axis, fixed by matching its saturation plateau above the gate-opening step to the saturated loading of the simulated HP framework, corresponding to a ∼ 15% increase. This accounts for the difference between the ideal, defect-free crystal represented in the simulation and the real sample, whose accessible capacity may be reduced by defects, partial crystallinity, and sample aging, sources of variability that are well documented for MOFs.^75^ The experimental isotherm exhibits a characteristic two step profile: the initial step at *p*/*p*_0_ ∼ 0.002 corresponds to the filling of sodalite cages in the LP conformation with subsequent adsorbate densification along a plateau near 400 cm^3^(STP)/g, which extends to *p*/*p*_0_ = ∼0.4. This behavior, which is typical for adsorption on microporous frameworks, is followed by an abrupt step in uptake to ∼540 cm^3^(STP)/g that is associated with the gate-opening transition to the HP conformation of a larger accessible pore volume.

**Figure 3:**
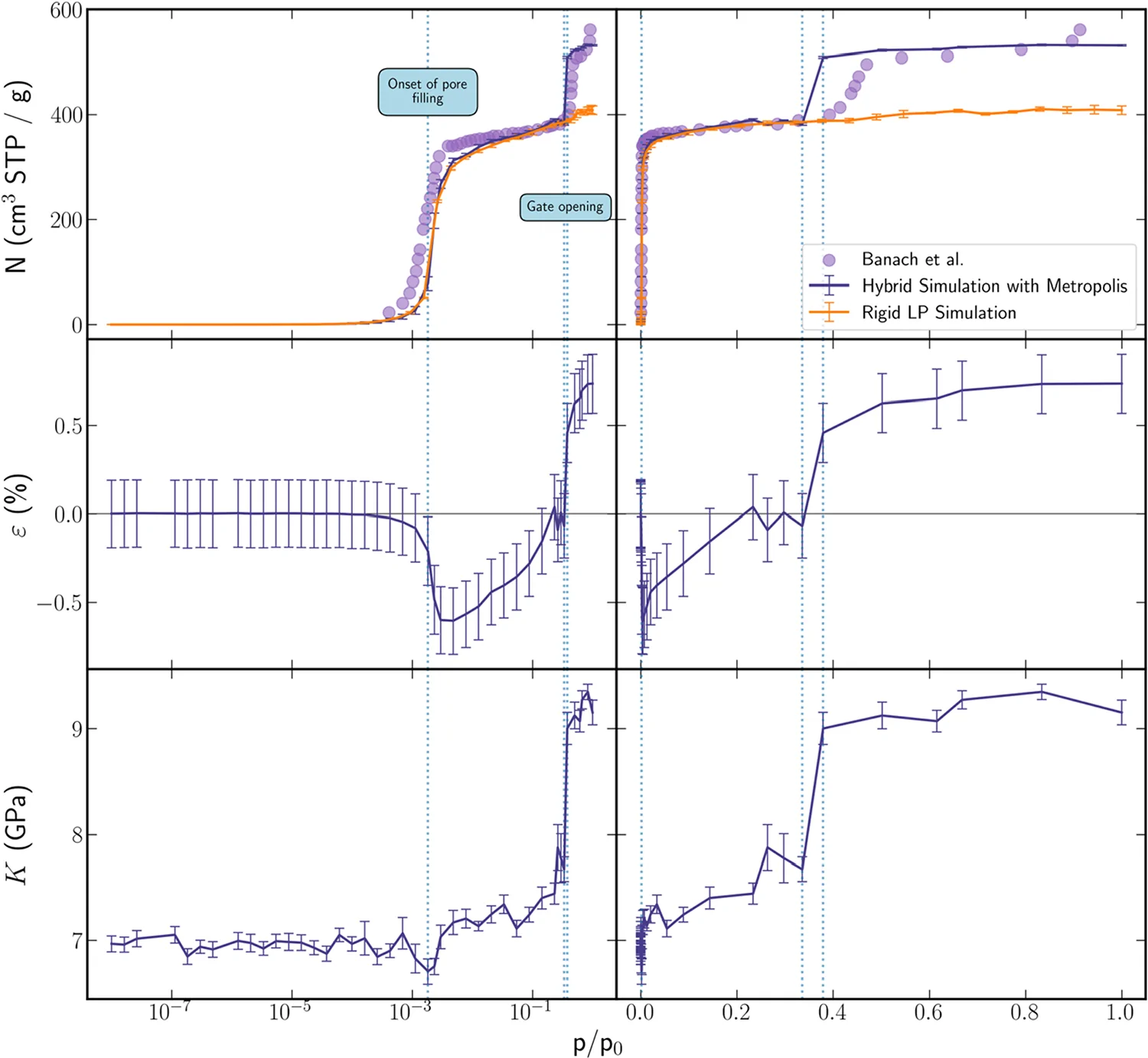
Argon adsorption on ZIF-8 at 87.3 K. Upper panels: Adsorption isotherms comparing experimental measurements (purple circles) with hybrid GCMC/MD simulations (blue lines with error bars) and rigid LP framework GCMC simulations (orange lines with error bars), shown on logarithmic (left) and linear (right) pressure scales. Middle panels: Volumetric strain from hybrid simulations. Lower panels: Bulk Modulus K derived from NPT-MD simulations.

The GCMC simulation on the rigid LP framework fairly accurately reproduces the low-pressure adsorption isotherm before the gate-opening transition and extends it to the maximum capacity of ∼410 cm^3^(STP)/g. Accounting for the framework flexibility by the hybrid GCMC/MD simulation allows us to capture the gate opening and quantitatively reproduce both the experimental pretransition plateau and post-transition uptake. The transition occurs close to the experimentally observed pressure (*p*/*p*_0_ ∼ 0.36 vs 0.39), and the capacities of closed- and open-gate structures match experimental observations. Noteworthy, at low pressures in the LP conformation, the simulated isotherms on rigid and flexible structures practically coincide across 7 orders of magnitude in pressure.

The hybrid simulation directly produces the volumetric strain isotherm shown in the middle panels of Figure 3. The strain isotherm in the LP framework is nonmonotonic: the framework contracts by ∼0.5% during pore filling as the guest molecules act as “molecular springs,” exerting a negative adsorption stress on the framework and pulling the host atoms inward. As loading increases and the adsorbed phase densifies, repulsive interactions with host molecules induce positive stress, and the framework expands along the adsorption isotherm plateau prior to gate opening. Such nonmonotonic deformation is typical for practically all microporous adsorbents; it reflects the competing mechanical influences of adsorbate-framework attraction (inducing contraction) and adsorbate–adsorbate repulsion during densification (causing expansion).^54,60,61,76–83^ Upon the LP-HP transition, the sample expands stepwise and then the volume gradually increases up to ∼0.7%, corresponding to the contrast of LP and HP framework volumes.

One of the advantages of the proposed method is the direct calculation of the variation of the framework compressibility during adsorption from the volume fluctuations, eq 2. The lower panels present the bulk modulus *K* = 1/β_*T*_. Because *K* is computed from the variance, its uncertainty cannot be obtained by simple error propagation on the mean volume and is sensitive to correlations between successive NPT-MD iterations. We therefore estimated error bars on *K* using a moving-block bootstrap (500 replicates, one standard deviation) of the per-iteration volume series, with block length set independently at each pressure to twice the integrated autocorrelation time of the corresponding volume trajectory. The framework maintains a relatively constant stiffness of ∼7 GPa at low pressures before the pore filling, matching well the reported experimental values of 6.5 GPa^26^ from mechanical compression and 7.7 GPa from Brillouin scattering.^84^ A very modest decrease is observed at the onset of pore filling, followed by stiffening during the densification plateau. Upon the gate-opening transition, the bulk modulus increases sharply to ∼9 GPa, showing an expected hardening of the loaded HP phase.

The use of the Metropolis acceptance rule is important for calculating the mechanical properties of loaded flexible frameworks. A direct comparison of simulations performed with and without the Metropolis acceptance rule is shown in Figures S1 and S2 of the Supporting Information, with acceptance fractions shown in Figure S3. While the adsorption isotherms are nearly indistinguishable between the two schemes, the strain and modulus isotherms are markedly different. Without the Metropolis filtering, the volumetric strain oscillates erratically across the full pressure range, including at low loadings where the framework should remain in a well-defined closed-gate state, and the bulk modulus fluctuates wildly between 3 and 11 GPa, reflecting the acceptance of mechanically inconsistent configurations. With the Metropolis filter in place, the strain isotherm exhibits a smooth characteristic nonmonotonic profile, and the bulk modulus is stable at the zero-loading close to the experimental LP value,^26,84^ with expected hardening of the structure following gate-opening. The acceptance rule is therefore essential for recovering thermodynamically consistent strain and compressibility. Because the test is evaluated once per iteration, it adds negligible cost, yet it determines whether the mechanical observables converge: for the same number of iterations, the volume fluctuations and hence the bulk modulus converge with the acceptance test and do not converge without it (Figures S1, S2). In addition, the longer NPT-MD runtime used in the proposed scheme may better accommodate slow framework rearrangements, such as linker reorientation, than the shorter MD moves attempted in the traditional HOMC scheme.^43^

This contrast identifies which properties are diagnostic of osmotic-ensemble sampling. The adsorption isotherm is not, since it depends primarily on the mean framework configuration and is reproduced regardless of whether the acceptance test is applied. The strain isotherm and compressibility are, because both are properties of the sampled volume distribution rather than of loading: the former follows from its mean, the latter from its fluctuations. The compressibility is the more demanding of the two, as a second-order property recovered from the volume fluctuations (eq 2) it is sensitive to the whole distribution rather than only its mean; such properties converge more slowly and are more demanding of the sampling scheme than the means from which the isotherm is built. This is why an adsorption isotherm can appear sound while the modulus obtained from the same trajectory is not.

The hybrid simulations demonstrate the importance of accounting for thermal fluctuations of the linkers. To elucidate the gate-opening mechanism, we analyzed the distribution of linker swing angles (θ_IM_) as defined by Tanaka et al.,^31^ which quantifies the rotation dynamics of the MeIM linkers about the N–Zn–N axis; θ_IM_ = 0 corresponds to the most probable linker orientation in the MD-equilibrated empty LP framework, which coincides with the crystallographic LP angle within the thermal fluctuations. Inherent thermal fluctuations around their equilibrium positions are shown in the upper panels of Figure 4, which present the probability distributions of θ_IM_ of the empty LP conformation (*p*/*p*_0_ = 10^–8^) and fully saturated HP conformation (*p*/*p*_0_ = 1).

**Figure 4:**
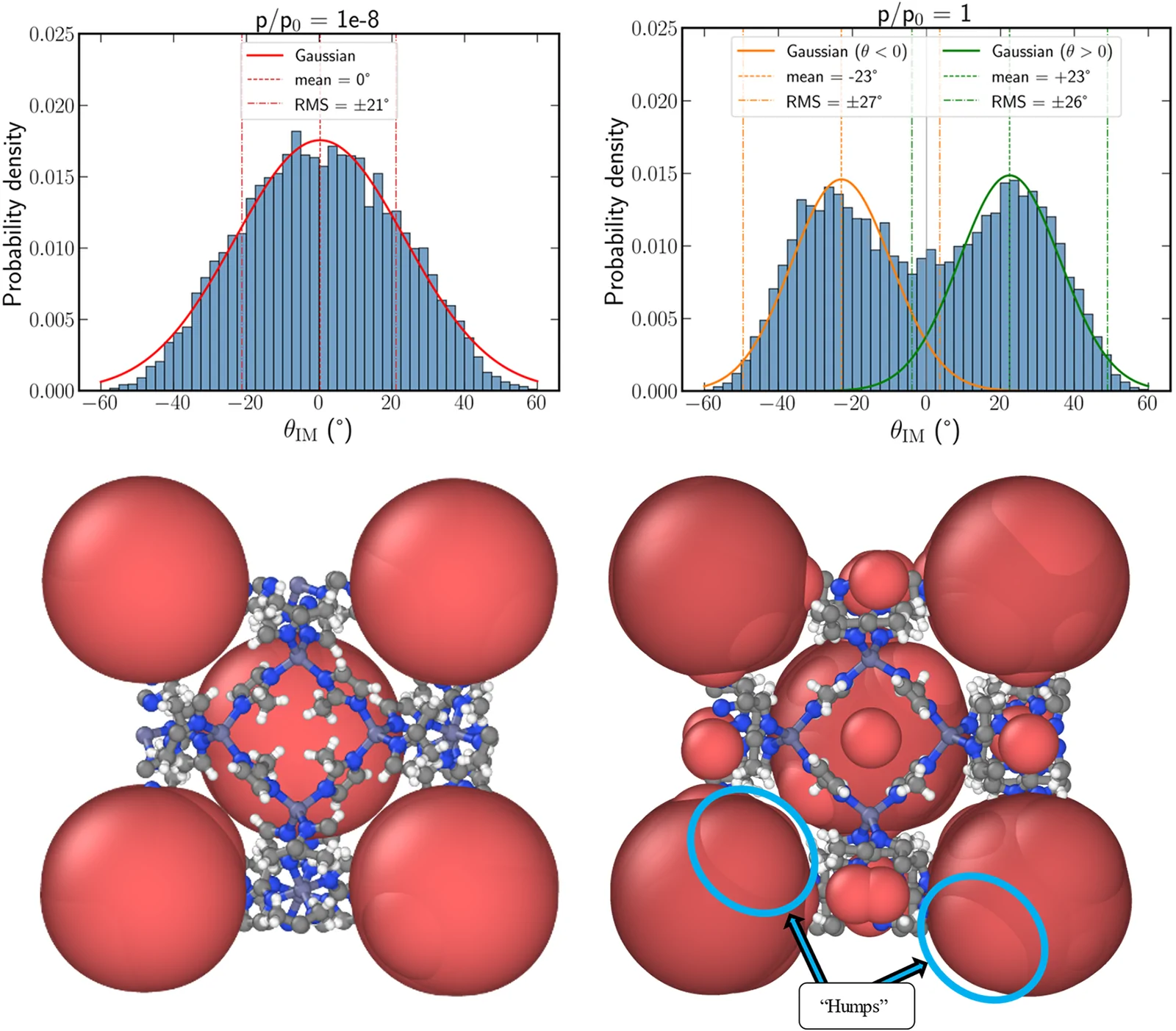
MeIM linker swing angle (θ_IM_) distributions and corresponding structural snapshots from hybrid GCMC/MD simulations. (top) Probability density distributions of θ_IM_ at *P* = 10^–8^ atm (left) and *P* = 1 atm (right). Solid lines indicate Gaussian distributions fitted to the data, dashed lines indicate the mean angle, and dotted lines indicate the median angle.. (bottom) Representative structural snapshots viewed along the [001] direction at *P* = 10^–8^ atm (left) and *P* = 1 atm (right), illustrating the transition from a closed-gate configuration to an open-gate configuration with rotated linkers adopting a propeller-like arrangement around the six- and 4-membered ring windows. Atom colors: Zn (gray-blue), N (blue), C (gray), H (white). Lower panels: Geometric visualization of probe-accessible pore space in ZIF-8 before and after the gate-opening transition. Accessible void regions (pink spheres) were calculated using Zeo++ ^85,86^ with an argon hard-sphere probe radius (0.17 nm) and are shown superimposed on the frame structure. (left) Structure at a reduced pressure of 0.34, prior to the gate-opening transition. (right) Structure at a reduced pressure of 0.55, after the transition.

Prior to the gate-opening transition, the angle distribution exhibits a unimodal profile centered near 0° with a sizable dispersion of 21°, characteristic to the thermal fluctuations of the closed-gate LP configuration. Following the gate-opening, the distribution becomes distinctly bimodal with symmetric peaks at approximately ± 23°. This bimodal character indicates that linkers have cooperatively rotated into two symmetry-equivalent open-gate orientations, consistent with the HP structure identified crystallographically. The magnitude of the linker reorientation agrees well with experimental estimates of 20–25°.^21,87^ The symmetric bimodal distribution arise from the alternating up-and-down configurations of adjacent linkers shown in Figure 1.

The specifics of the gate-opening transformation are visualized by structural snapshots in Figure 4 (lower panels). In the closed-gate configuration, linkers adopt a relatively uniform orientation around the six-membered ring windows. Following the gate-opening, linkers rotate and align into an arrangement that simultaneously enlarges the six-membered rings connecting adjacent cages and opens the four-membered ring windows between cages. Geometric pore characterization using Zeo++ ^85,86^ with an argon probe (radius 0.17 nm) demonstrates these changes, as shown by visualizing the probe-accessible pore volume using pink spheres: the six-membered ring apertures exhibit increased accessible volume, visible as “humps” extending from the main cage spheres, while four-membered rings, previously too narrow, now accommodate probe molecules.

The gate-opening transition from LP to HP conformation is associated with cooperative adsorbate reorganization, as shown in Figure 5 by representative simulation snapshots of adsorbed argon distributions. Prior to the transition (left), argon molecules exhibit relatively disordered packing within the sodalite cages and through the six-membered rings. Following the transition (right), argon adopts a markedly more ordered arrangement, consistent with experimental observations showing adsorbate repacking.^34^ The ordered structure of Ar in the HP structure shown in Figure 5 is similar to the Ar distributions reconstructed by in situ high-pressure crystallography and simulated by GCMC in the rigid HP structure by Hobday et al.^25^ Together with the quantification of the linker fluctuations in Figure 4, our simulations demonstrate the dynamics of coupling structural transformations in the guest and host phases. This structural ordering enables more efficient molecular packing, contributing to the stepped increase in uptake capacity.

**Figure 5:**
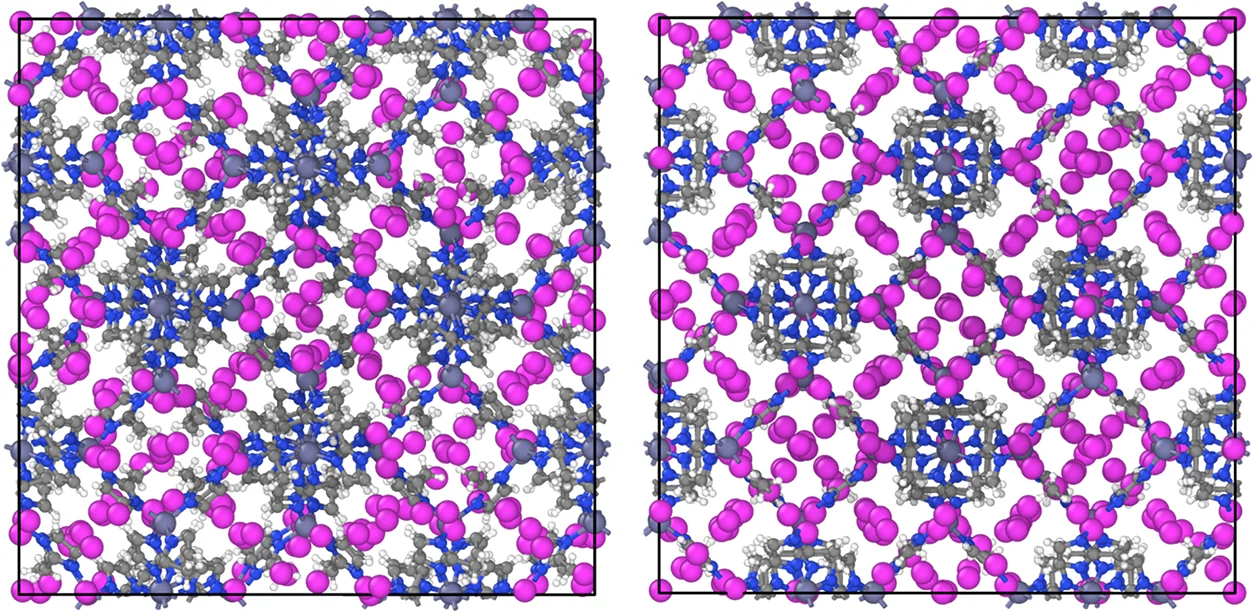
Representative simulation snapshots of the ZIF-8 supercell with adsorbed argon at 87.3 K (left) before the gate-opening transition at a reduced pressure of 0.34 (right) and after the transition at a reduced pressure of 0.55. The ZIF-8 framework is shown with carbon (gray), nitrogen (blue), zinc (gray-blue), and hydrogen (white) atoms, while adsorbed argon molecules are represented as purple spheres.

## Conclusions

We have presented an iterative hybrid GCMC/MD methodology that explicitly enforces thermodynamic consistency through a Metropolis acceptance criterion in the osmotic ensemble. The scheme partitions the trial moves between a GCMC substage that samples adsorbate number in the fixed framework and a combined NVT & NPT-MD substage that samples fluctuations of the framework configuration and volume at a fixed number of adsorbate molecules. By executing these two substages sequentially with the respective Metropolis grand canonical (μ,*P*,*T*) and isothermal–isobaric ensemble (*N P*,*T*) acceptance criteria in a single combined trial move, the scheme preserves the detailed balance with respect to the osmotic (*N*_*f*_,μ,*P*,*T*) ensemble.

Applied to argon adsorption on ZIF-8 at 87.3 K, the method quantitatively reproduces the experimental isotherm below and above the gate opening transition, capturing the transition pressure, the two-step profile, and the relative magnitudes of the LP and HP adsorption capacities. In addition to the adsorption isotherm, the proposed method enables monitoring and quantification of adsorption-induced framework dynamics that are inaccessible in rigid-framework simulations. Three main findings are worth special note. First, before the transition, the LP structure exhibits a nonmonotonic strain isotherm characteristic of microporous adsorbents, with contraction at low loadings followed by expansion after the pore filling as the adsorbate densifies, and subsequent sharp expansion upon the transition to HP structure. Second, the bulk modulus extracted from the volume fluctuations matches the mechanical compression and Brillouin scattering measurements in the empty LP phase. During adsorption, the structure stiffens upon pore filling and guest molecule densification with a sharp hardening upon the transition to the saturated HP structure. Third, during the LP-HP transition, the linker-swing angle distribution evolves from a unimodal distribution in the LP phase to a symmetric bimodal distribution in the HP phase, in agreement with crystallographic data, that provides direct molecular-level evidence for the cooperative propeller-like reorganization of the six- and four-membered ring windows. This framework reorganization is accompanied by pronounced ordering of the guest molecule packing.

The Metropolis acceptance probability provides a rigorous filter that retains only mechanically and thermodynamically consistent configurations, in contrast to previous iterative MC/MD protocols that rely on an empirical convergence of loading and volume. A direct comparison with simulations performed without the Metropolis filter demonstrates that the acceptance criterion is essential for recovering thermodynamically meaningful mechanical properties (Figures S1 and S2). Without filtering, the adsorption isotherm remains qualitatively consistent, but the strain and modulus isotherms fluctuate erratically, reflecting accumulation of mechanically inconsistent configurations. The Metropolis criterion suppresses these unphysical fluctuations and yields reasonably smooth isotherms. This observation reconciles the recent concerns raised by Ghoufi^63^ regarding the ergodic properties of hybrid MD/MC schemes: statistical ergodicity, and hence the reliable prediction of equilibrium properties, is preserved provided detailed balance is enforced in the appropriate ensemble.

A distinguishing feature of the proposed scheme is that *a priori* assumptions about the transition pass or post-transition structure are not required. The framework transformation emerges spontaneously from the coupled equilibration of adsorbate loading and framework configuration, with linker reorientation, pore-aperture enlargement, adsorbate repacking, and framework expansion, all developing self-consistently in response to the rising adsorption stress induced by guest molecules. Provided robust flexible force fields for the host–host framework and host–guest interactions, the method offers a general predictive route to adsorption-induced structural transitions in flexible nanoporous materials. Although we present here the simplest implementation limited to the isotropic volumetric deformation, its advancement to account for anisotropic framework deformation by using the isostress osmotic ensemble is straightforward.

The proposed methodology is readily transferable to other flexible MOFs exhibiting breathing, gate-opening, or subnetwork thermal transitions, as well as to other porous materials such as microporous polymers and carbons. Extension of the method includes multicomponent adsorption and mixture-dependent structural transitions, as well as temperature-dependent and hysteretic transformations.

## Supplementary Material



## Data Availability

The python controller script and LAMMPS input files used in this work are available at the following repository: https://github.com/njcorrente/pySIMAID

## Notes

Comparison of isotherms with and without Metropolis filtering (PDF)
