# Supplementary material for: A Thermodynamically Consistent Approach to Molecular Simulations of Adsorption-Induced Deformation and Structural Transitions in MOFs
Source: J Chem Theory Comput. 2026 Oct 7;22(20):10636–46. doi: 10.1021/acs.jctc.6c01243 (PMC13718537; doi:10.1021/acs.jctc.6c01243)
Supplement: Supplementary file 1 [file ct6c01243_si_001.pdf]

A Thermodynamically Consistent Approach to Molecular Simulations of Adsorption-Induced Deformation and Structural Transitions in MOFs

Nicholas J. Corrente, Kaelyn Chang, Muhtasim Noor, and Alexander V. Neimark
Department of Chemical and Biochemical Engineering, Rutgers University, USA
Email: aneimark@rutgers.edu; Web: http://neimark.rutgers.edu/

Supporting Information

In Figures S1 and S2, we demonstrate advantages of the proposed osmotic ensemble hybrid MC-MD simulation scheme comparing the simulation results with and without the Metropolis sampling.

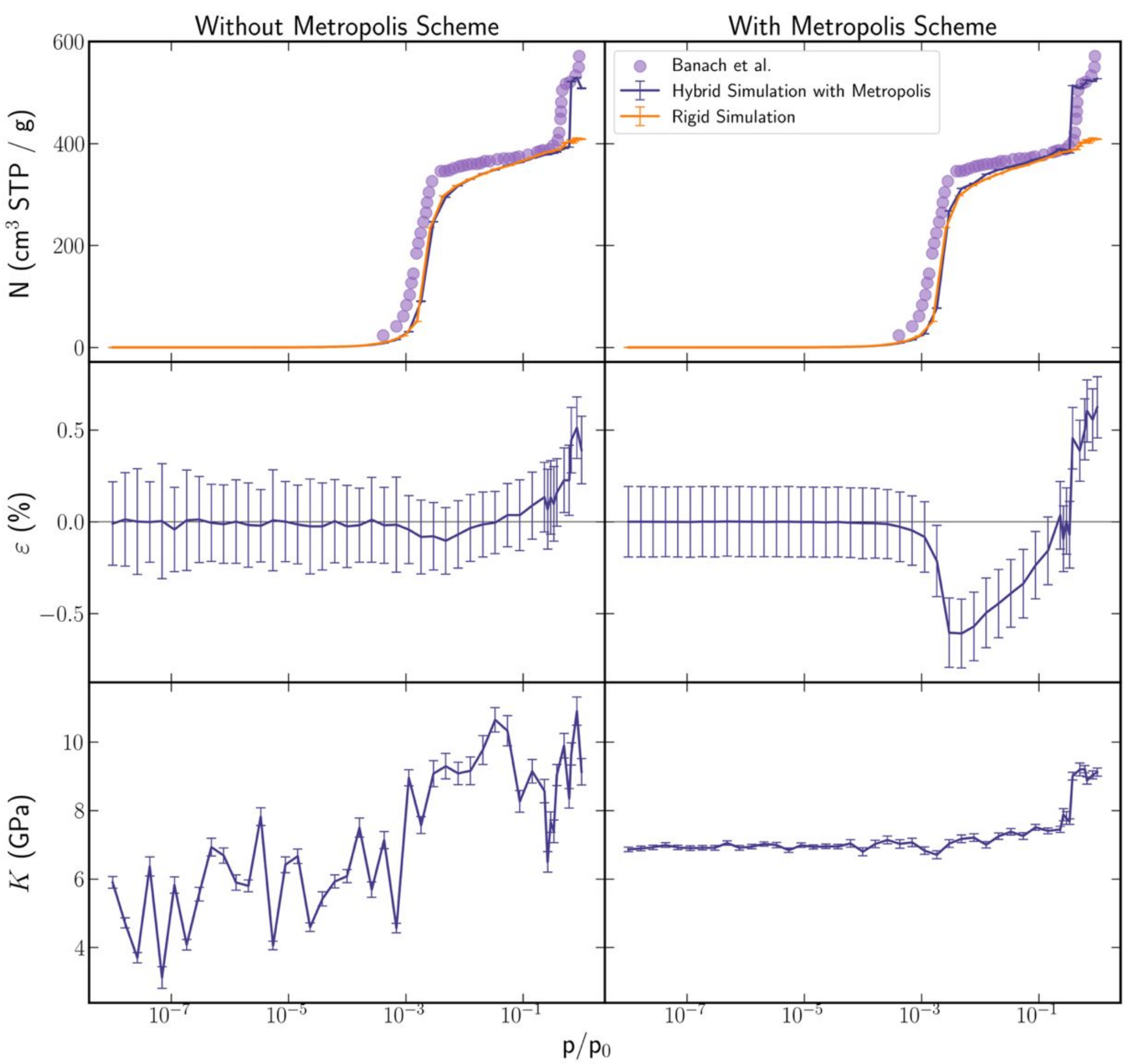

Figure S1: Comparison of hybrid GCMC/MD simulations with (right) and without (left) Metropolis acceptance in the osmotic ensemble for argon adsorption on ZIF-8 at 87.3 K

Figure S1 compares simulations performed with (right panels) and without (left panels) the Metropolis acceptance criterion. Although the adsorption isotherms in both schemes match well, the strain and modulus isotherms differ greatly, with the simulations without Metropolis filtering showing several erroneous behaviors. The volumetric strain oscillates erratically across the entire pressure range with no clear correlation to loading, showing large variations even at low pressures where the framework should remain in a well-defined closed-gate state. Similarly, the bulk modulus K fluctuates wildly between 3 and 11 GPa throughout the isotherm, reflecting the acceptance of mechanically inconsistent configurations. These fluctuations are artificial, arising from the absence of thermodynamic filtering.

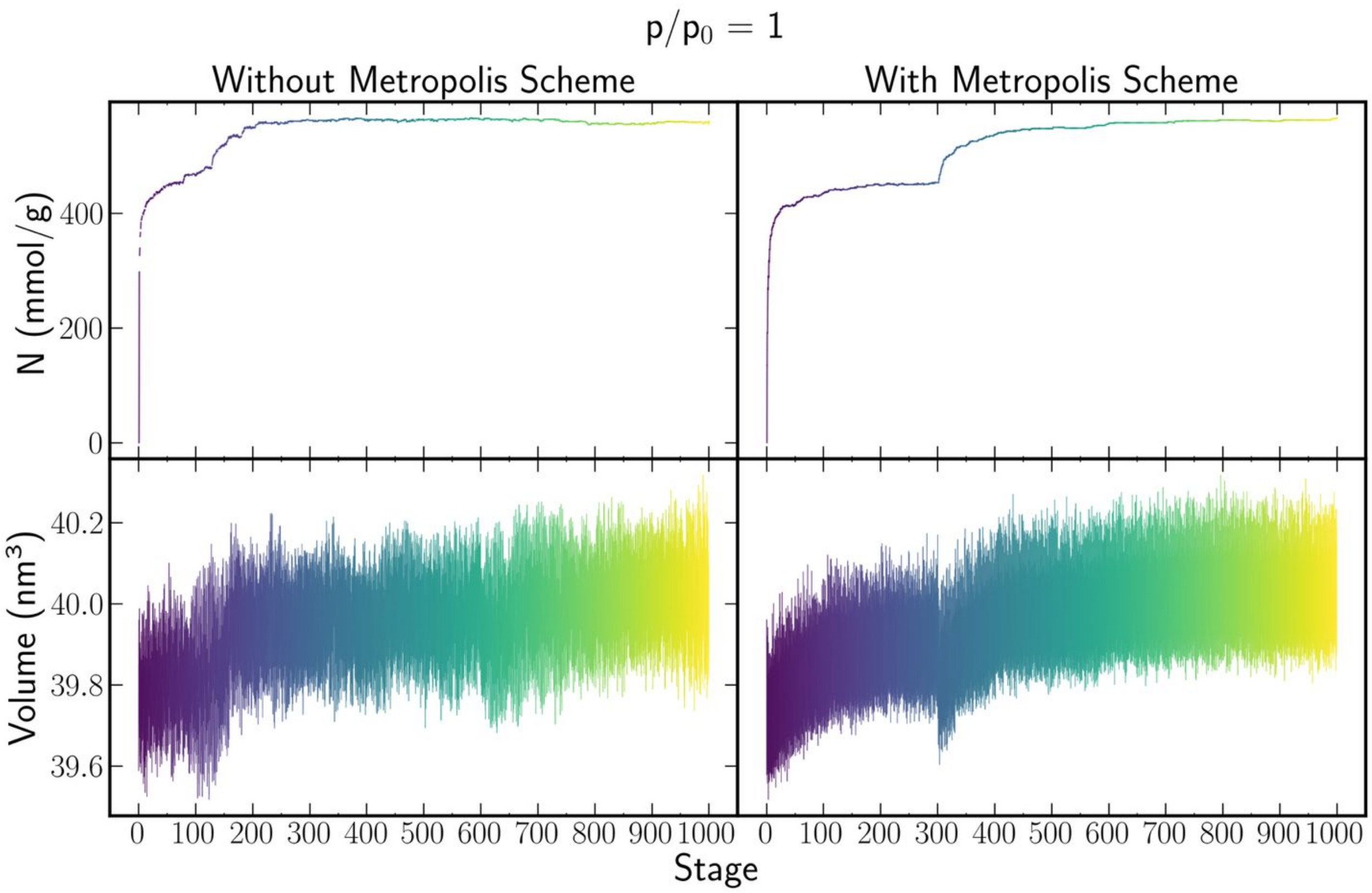

Figure S2: Convergence of the loading (top) and framework volume (bottom) as a function of iteration stage at the highest pressure point ($p/p_0 = 1$) of the isotherms shown in Figure S1, without (left) and with (right) the Metropolis acceptance criterion. Note that the simulation starts from the empty LP conformation. The gate opening transition occurs after ~ 300 iteration stages. The fluctuations are stabilized after ~ 700 stages.

Figure S2 shows the stage-by-stage evolution of the adsorbate loading and framework volume at $p/p_0 = 1$, corresponding to the final pressure point of the isotherm in Figure S1. Without the Metropolis filter (left column), the loading climbs gradually and somewhat irregularly toward its plateau value, with the volume fluctuations broadening progressively as configurations from successive NPT-MD segments are accepted unconditionally. With the Metropolis filter (right column), the loading exhibits a sharper, more clearly defined transition between the pre-gate-opening plateau in LP conformation and the saturated state in HP conformation near stage 300; after ~700 stages both $N$and Vsettle into well-defined averages with stable fluctuations that are used for calculating the mean squire fluctuations.

The narrower and more uniform volume fluctuations in the filtered trajectory reflect the rejection of mechanically inconsistent configurations and underlie the strain and modulus isotherms reported in the main text. The unfiltered trajectory, by contrast, retains such configurations throughout and produces the erratic mechanical response shown in Figure S1.

Because the filtered trajectory resolves the transition cleanly, a feature becomes visible: immediately before the jump in loading, the volume undergoes a brief but pronounced contraction. As the rising loading drives the MeIM linkers away from their closed-gate equilibrium, the linkers pass through intermediate orientations in which the framework is transiently contracted relative to either the closed or fully open state. Once the linkers reach their new aligned equilibrium with the gate open, the framework relaxes outward and the volume jumps to its expanded HP value, accompanied by the step in loading.

To conclude, the Metropolis criteria for transfers between the MD and MC substages significantly reduces the loading and especially volume fluctuations. While the Metropolis criterion is not essential for simulating the adsorption isotherm, the strain isotherm and especially the compressibility are defined with an excellent accuracy unachievable without this filter.

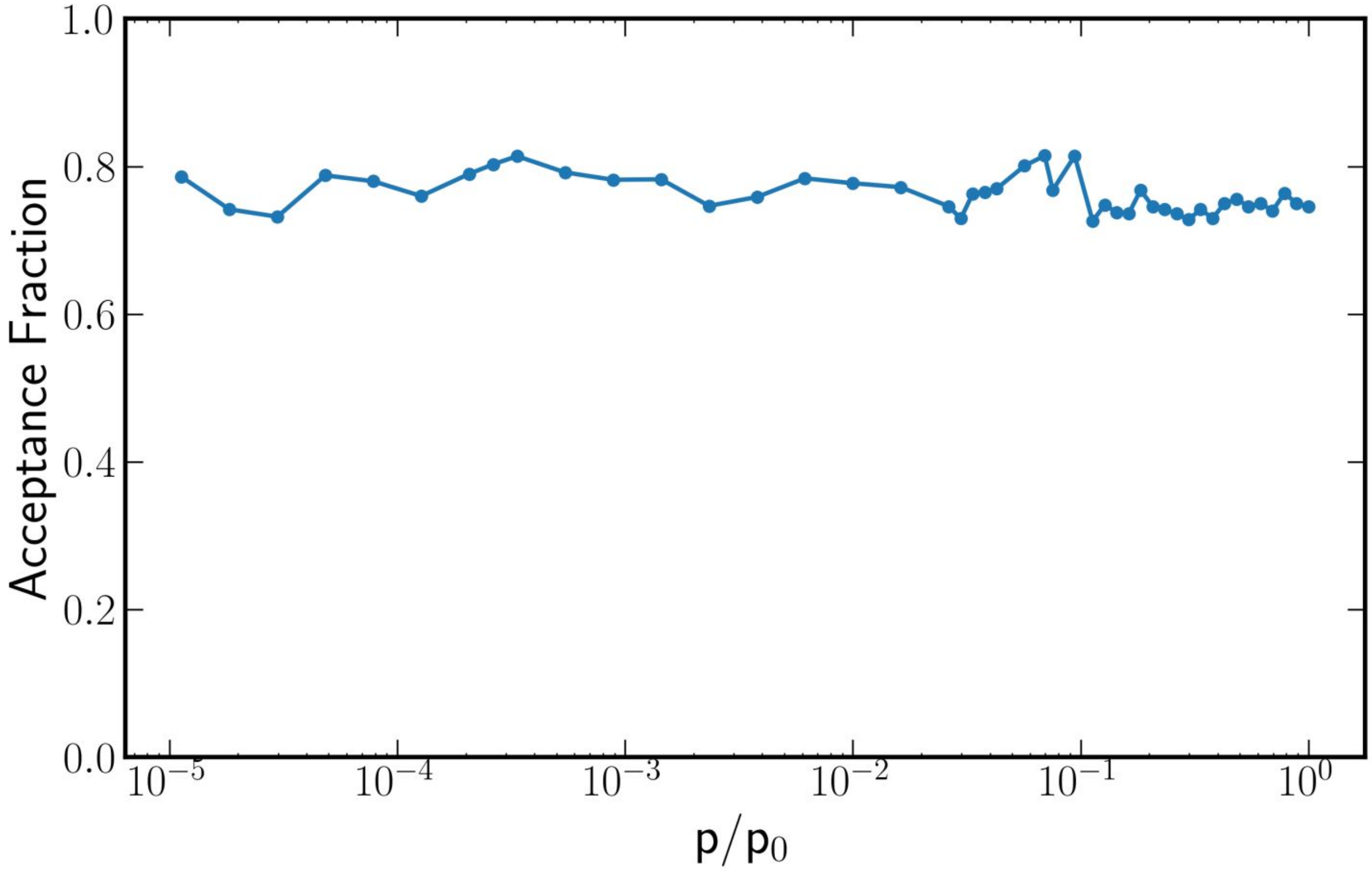

Figure S3: Acceptance fraction of the hybrid NPT-MD volume moves from Figure 3 of the main text as a function of relative pressure using the proposed scheme with the Metropolis acceptance criterion.

Figure S3 shows the acceptance fraction at each pressure considered, defined as the number of accepted volume moves divided by the number of attempted volume moves. Summary statistics are given in Table S1, with the data partitioned at the gate-opening transition.

Table S1: Acceptance statistics of the acceptance fractions shown in Figure S3.

**Pressure Range**	Mean	Standard Deviation
Full Range	0.762	0.025
Prior to Gate Opening	0.775	0.024
Post Gate Opening	0.744	0.011

Two features are worth noting. First, the acceptance fraction does not degrade in the vicinity of the transition where the framework undergoes its largest volume change and where sampling difficulties would most likely exist. Second, the mean slightly decreases following the gate-opening transition, which corresponds to a sharp increase in adsorbate loading. The additional degrees of freedom added by the adsorbate atoms is expected to reduce the acceptance rate.
